# Investigation on the hereditary basis of colorectal cancers in an African population with frequent early onset cases

**DOI:** 10.1371/journal.pone.0224023

**Published:** 2019-10-24

**Authors:** Leolin Katsidzira, Anna Vorster, Innocent T. Gangaidzo, Rudo Makunike-Mutasa, Dhiren Govender, Simbarashe Rusakaniko, Sandie Thomson, Jonathan A. Matenga, Raj Ramesar

**Affiliations:** 1 Department of Medicine, College of Health Sciences, University of Zimbabwe, Harare, Zimbabwe; 2 MRC/UCT Research Unit for Genomic and Precision Medicine, Division of Human Genetics, Institute of Infectious Diseases and Molecular Medicine, Faculty of Health Sciences, University of Cape Town, Cape Town, South Africa; 3 Central Analytical Facility (CAF), DNA Sequencing Unit, Stellenbosch University, Stellenbosch, South Africa; 4 Department of Histopathology, College of Health Sciences, University of Zimbabwe, Harare, Zimbabwe; 5 Division of Anatomical Pathology, Faculty of Health Sciences, University of Cape Town, and National Health Laboratory Service Groote Schuur hospital, Cape Town, South Africa; 6 Department of Community Medicine, College of Health Sciences, University of Zimbabwe, Harare, Zimbabwe; 7 Division of Gastroenterology, Department of Medicine, University of Cape Town, Cape Town, Groote Schuur Hospital, Cape Town, South Africa; CNR, ITALY

## Abstract

**Background:**

Approximately 25% of colorectal cancer patients in sub-Saharan Africa are younger than 40 years, and hereditary factors may contribute. We investigated the frequency and patterns of inherited colorectal cancer among black Zimbabweans.

**Methods:**

A population-based cross-sectional study of ninety individuals with a new diagnosis of colorectal cancer was carried out in Harare, Zimbabwe between November 2012 and December 2015. Phenotypic data was obtained using interviewer administered questionnaires, and reviewing clinical and pathology data. Cases were screened for mismatch repair deficiency by immunohistochemistry and/or microsatellite instability testing, and for *MLH1*, *MSH2* and *EPCAM* deletions using multiplex ligation-dependent probe amplification. Next generation sequencing using a 16-gene panel was performed for cases with phenotypic features consistent with familial colorectal cancer. Variants were assessed for pathogenicity using the mean allele frequency, phenotypic features and searching online databases.

**Results:**

Three Lynch syndrome cases were identified: *MSH2 c*.*2634G>A* pathogenic mutation, c.(1896+1_1897–1)_(*193_?*)del *, and one fulfilling the Amsterdam criteria, with *MLH1* and *PMS2* deficiency, but no identifiable pathogenic mutation. Two other cases had a strong family history of cancers, but the exact syndrome was not identified. The prevalence of Lynch syndrome was 3·3% (95% CI 0·7–9·4), and that of familial colorectal cancer was 5·6% (95% CI, 1·8–12·5).

**Conclusions:**

Identifying cases of inherited colorectal cancer in sub-Saharan Africa is feasible, and our findings can inform screening guidelines appropriate to this setting.

## Introduction

The incidence of colorectal cancer is gradually rising in some countries in sub-Saharan Africa, including Kenya, Nigeria, and Zimbabwe.[1] In Zimbabwe, the incidence of colorectal cancer has been rising steadily, averaging 4% annually since 1991.[2] This rising incidence is probably due to improvements in diagnosis, and to changes in the prevalence of known risk factors.[1] These factors include urbanisation, diabetes mellitus, schistosomiasis, and shifts from traditional dietary patterns, which are associated with colorectal cancer among black Zimbabweans.[3, 4] There is circumstantial evidence that familial predisposition plays a prominent role in colorectal cancer across Africa, but the degree and patterns have not been characterised.

Approximately 1 in 4 individuals with colorectal cancer in sub-Saharan Africa is under the age of 40 years.[1] In general, young people with colorectal cancer are more likely to have a pathogenic germline mutation.[5, 6] Thus, it has been hypothesised that the frequency of hereditary colorectal cancers, particularly Lynch syndrome, is high in sub-Saharan Africa.[7] This is supported by the high frequency of histological features associated with Lynch syndrome (mucinous and signet ring cell morphology), and of mismatch repair protein deficiency in colorectal cancers in this region.[7–9] The earlier age of onset of colorectal cancer among African-Americans also provides additional, indirect evidence of an intrinsic genetic predisposition among people of African ancestry.[10]

While this theory is plausible, there is lack of solid supporting evidence. Previous studies reported the frequency of mismatch repair deficiency in archived tissue, without further, comprehensive analysis.[7, 9] Phenotypic data, in particular family history, was often inadequate, and the causative mutations were not characterised. The increasing availability of next generation sequencing platforms in a few academic centres in Africa, provides an opportunity to bridge this gap. Therefore, we investigated the frequency and mutation spectrum of hereditary colorectal cancers in a prospective, population-based cohort of black Zimbabwean patients.

## Materials and methods

### Study population

Newly diagnosed cases of colorectal cancer were prospectively recruited in Harare, Zimbabwe, between November 2012 and December 2014, as previously described.[3] They were recruited from hospitals, clinicians, and pathology laboratories in both public and private practice in Harare. The cases, who mainly came from the northern two thirds of Zimbabwe, were representative of the overall black population. Pathology specimens from this region are processed at one of four laboratories in Harare, allowing accurate case tracing. A network of pathologists, and clinicians was consequently established for this purpose. Ethical approval was obtained from the institutional review boards of the University of Zimbabwe, the University of Cape Town, and the Medical Research Council of Zimbabwe. The study complied with the Helsinki declaration and its later amendments.

### Data collection

The participants were recruited within six months of a histologically confirmed diagnosis of colorectal cancer. After informed consent, phenotypic data was obtained using interviewer-administered questionnaires covering demographic variables, and detailed personal, and family history of cancer. Data on tumour pathology (location, morphology, grading and presence of synchronous neoplasia) were obtained from pathology and endoscopic reports.

Venous blood was drawn for germline DNA, and paraffin-embedded tumour blocks were obtained from pathology laboratories. Initial screening for Lynch syndrome was performed using immunohistochemistry and/or microsatellite instability testing. Immunohistochemistry was used as a parallel method of assessing mismatch repair deficiency, and to possibly indicate the mutated gene. Screening for germline *MLH1*, *MSH2* and *EPCAM* deletions was performed regardless of the mismatch repair status using multiplex ligation-dependent probe amplification (MLPA). A phenotype-guided strategy was then used to select cases for next generation germline sequencing. The phenotypic features considered included family history of cancer and colorectal cancer, prior history of cancer, age < 40 years, mucinous or signet ring morphology, presence of synchronous tumours, polyposis, mismatch repair-protein status and *BRAF V600E* result.

### Screening for Lynch syndrome

Microsatellite instability (MSI) testing was performed on paired germline and tumour DNA samples using the Promega MSI Analysis System version 1.2 (Promega Corporation, Madison, Wisconsin, United States). Germline DNA was obtained from peripheral blood, or normal tissue on tumour slides using standard techniques. Fluorescently-labelled primers were used for co-amplification of seven markers; five mononucleotide (BAT-25, BAT-26, NR-21, NR-24 and MONO-27), and two pentanucleotide (Penta C and Penta D) repeat markers. The PCR products were separated by capillary electrophoresis (Applied Biosystems 3130 Genetic Analyzer), and the output analysed with GeneMapper version 4.0 software (Applied Biosystems; Foster City, CA, USA). The results were interpreted as follows: MSI at 2 or more mononucleotide loci, MSI-high (MSI-H); MSI at a single mononucleotide locus, MSI-Low (MSI-L) and no instability at any of the loci, microsatellite stable (MSS).

Immunohistochemistry was performed using anti-*MSH2* and anti-*PMS2* mouse monoclonal antibodies (Cell Marque; Rocklin, California, USA), and anti-*MLH1* and anti-*MSH6* mouse monoclonal antibodies (Ventana Medical Systems; Tucson, Arizona, USA) on formalin-fixed, paraffin-embedded tissue sections. Tissue sections with normal colonic mucosa adjacent to the tumour were selected for immunohistochemistry. The normal areas acted as internal positive controls, while external control positive controls were also included (normal colonic mucosa). A Ventana Benchmark XT autostainer and the Opti-view detection system (Ventana Medical Systems; Tucson, Arizona, USA) were used, following the manufacturers’ protocol. The stained slides were interpreted by an experienced pathologist (DG).

All cases that were *MLH1* protein deficient, with a high level of microsatellite instability (MSI-H) were sequenced for the *BRAF V600E* mutation. The following primers were used: Forward, 5'-CTACTGTTTTCCTTTACTTACTACACCTCAGA-3'; and reverse, 5'-ATCCAGACAACTGTTCAAACTGATG-3'. Sequencing was performed using BigDye terminator v3.1 cycle sequencing kit (Thermofisher Scientific, Wilmington, USA) following standard protocols.

MLPA for *MLH1*, *MSH2* and *EPCAM* germline deletions was performed using the SALSA MLPA kit (MRC-Holland, Amsterdam, Netherlands) according to the manufacturer’s protocol. Briefly, germline DNA was denatured, followed by hybridisation with MLPA probes, ligation and amplification. The amplified products were visualised using capillary electrophoresis, and the output analysed using the Coffalyser.Net software (https://www.mlpa.com).

### Next generation sequencing using a multigene panel

Next generation sequencing of germline DNA from peripheral blood was performed on selected cases using a custom panel of 16 colorectal cancer genes (*MUTYH*, *EPCAM*, *MSH2*, *MSH6*, *MLH1*, *PMS2*, *FBX011*, *APC*, *BMPRIA*, *KLLN*, *PTEN*, *POLE*, *POLD1*, *TP53*, *SMAD4*, and *STK11*). DNA quality assurance was performed using agarose gel electrophoresis and spectrophotometry (Nano-Drop 1000) (ThermoFisher Scientific, Wilmington, USA). DNA quantification was performed using real time PCR with the RNase P assay. DNA dilutions and library preparation were performed on the Ion Chef using the DL8 kit (ThermoFisher Scientific, Wilmington, USA), followed by template preparation and sequencing on the Ion Chef personal genome machine as per manufacturer’s protocol. The output was analysed using the Ion Reporter ((ThermoFisher Scientific, Wilmington, USA). Variants with a mean allele frequency< 0.01 were considered potentially pathogenic, and cross-referenced with online databases, including Clin-Var (https://www.ncbi.nlm.nih.gov/clinvar/), InSiGHT (https://www.insight-group.org/variants/databases/), and LOVD (http://www.lovd.nl/3.0/home). Phenotypic characteristics, particularly family history, immunohistochemistry and MSI status were considered in evaluating pathogenicity.

### Statistical methods

Descriptive statistics including percentages (with 95% confidence intervals), mean (with standard deviations), and median (with inter-quartile range) were used to summarise data. The statistical analysis was performed using Stata MP Version 12.0® (College Station, Texas, USA).

## Results

**Fig 1:**
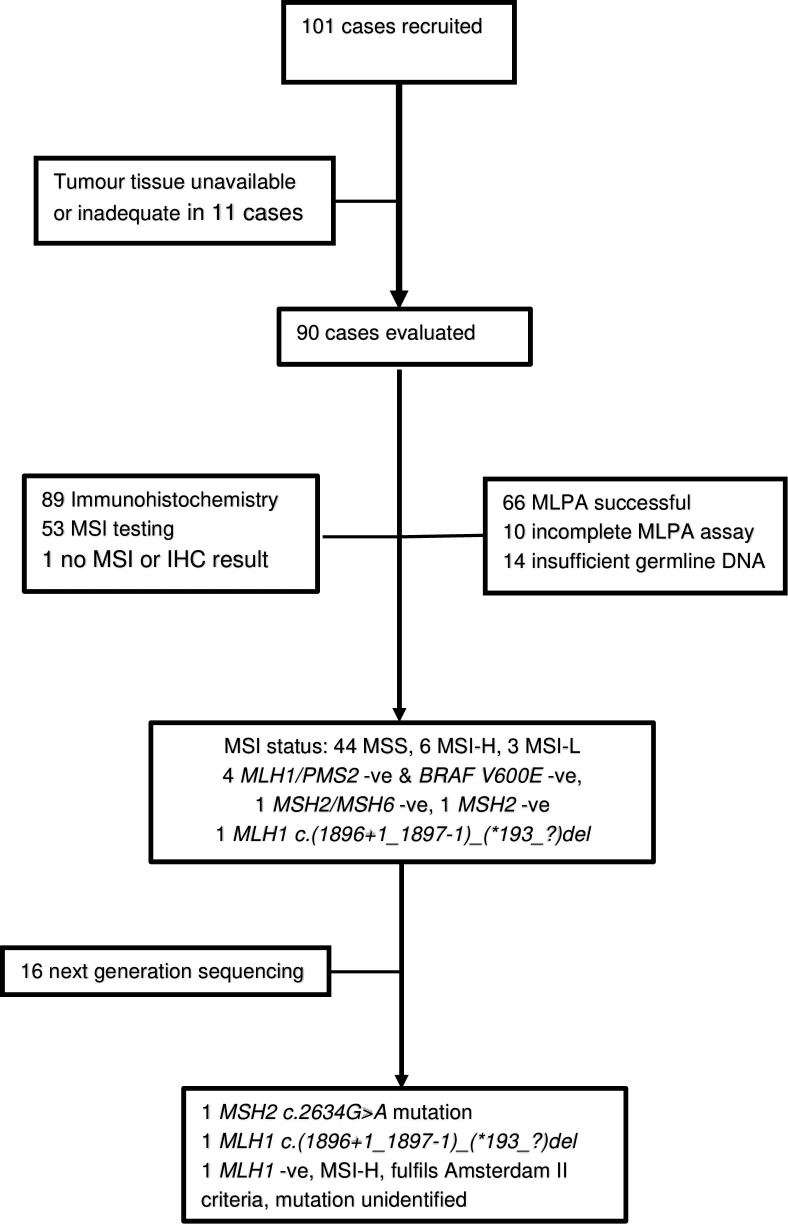
Study flowchart describing the laboratory process for evaluating cases for inherited colorectal cancer (-ve–negative).

A total of 101 black Zimbabwean patients with newly diagnosed colorectal cancer were recruited in Harare, between November 2012 and December 2015. Complete analysis for hereditary colorectal syndromes was performed in 90 cases (Fig 1). The mean age for these cases was 52·3 years, with 20 (22%) under 40 years of age (Table 1). Sixty-eight cases (75·6%), had tumours distal to the splenic flexure (i.e. left-sided), including 49 (54%), that were in the rectum. There were 14 (16%) mucinous adenocarcinomas and 8 (9%) signet ring cell carcinomas. One case fulfilled the Amsterdam II criteria for Lynch syndrome.

**Table 1:** Demographic and clinical characteristics in the ninety cases.

Variable	Frequency n (%) or Mean (SD)
**Mean age (Years)**	52·3(14·9)
**Age Category (Years)**	
< 40	20 (22·2)
41–50	19 (21·1)
>50	51 (56·7)
**Females**	46 (5·1)
**Tumour site**	
Proximal colon	18 (20·0)
Distal colon	19 (21·1)
Rectum	49(54·5)
Synchronous hepatic flexure and rectum	1(1·1)
Colon, not specified	3(3·3)
**Histology**	
Adenocarcinoma	68(75·6)
Mucinous adenocarcinoma	14(15·5)
Signet ring cell carcinoma	8(8·9)
**Cancer in 1**^**st**^ **degree relatives**	18(20)
**Colorectal cancer in 1**^**st**^ **degree relatives**	2
**Personal history of cancer**	3 (3·3)
**Amsterdam II Criteria**^†^^[^^11^^]^	1
**At least one Bethesda criterion present**^‡^^[^^12^^]^	45 (50)

†3 or more family members with a Lynch tumour, 2 successive generations, 1 or more Lynch related tumour before the age of 50 years.

‡CRC at < 50 years, synchronous or metachronous CRC or other Lynch tumours, MSI-H histology at age < 60 years, appropriate family history.

### The frequency and patterns of mismatch repair deficiency

Mismatch repair status was available in 89 cases; 53 from MSI and immunohistochemistry, and 36 from immunohistochemistry alone (Table 2). There were no mismatch repair results in one case, but he was selected for next generation sequencing because of ‘young age’, and abnormal MLPA result. The MSI status in the 53 cases were as follows: MSS, 44 (83%); MSI-H, 6 (11·3%) and MSI-L), 3 (5·7%). Mismatch repair protein deficiency on immunohistochemistry was present in 6 (6·7%) of the 89 cases. Four were *MLH1* and *PMS2* deficient (and *BRAF V600E* negative), one was MSH*2* and *MSH6* deficient, and one had isolated *MSH2* deficiency. In the 53 cases where both immunohistochemistry and MSI results were available, there was concordance in all, but one case (98·1% concordance) (Table 2).

**Table 2:** Mismatch repair status in 89 cases of colorectal cancer and the likelihood of Lynch syndrome.

MSI status (n)	Protein expression				Interpretation
	MLH1	MSH2	MSH6	PMS2	
MSS (44)	+	+	+	+	Not Lynch
MSI-H (1)	+	+	+	+	Discordant result
MSI- H (1)	+	-	-	+	Lynch likely
MSI-H (4)	-	+	+	-	Lynch or sporadic
MSI-L (3)	+	+	+	+	Not Lynch
Unknown (35) †	+	+	+	+	Not Lynch
Unknown (1)	+	-	+	+	Likely Lynch

†Incomplete MSI testing in 31, no normal tissue in 4.

### The frequency and patterns of pathogenic germline variants identified by next generation sequencing

Next generation sequencing was performed in 16 cases summarised in Table 3. Two cases had a history of colorectal cancer in 1^st^ degree relatives, one of whom met the Amsterdam criteria for Lynch syndrome. One case of Lynch syndrome was identified on next generation sequencing, a 48-year-old woman with a rectal tumour, and a pathogenic variant in *MSH2*, (NM_000251.2, *c*.*2634G>A*, *p*.*Glu878 =*), but no obvious family history of colorectal cancer (Table 4). Immunohistochemistry was consistent with a pathogenic variant, with loss of expression of *MSH2*. This variant has been shown to be pathogenic in a Spanish population, and causes splicing aberration leading to a truncated protein.[13, 14] There were no germline pathogenic variants identified in any of the 16 genes in the other 15 cases.

**Table 3:** Characteristics of the 16 cases selected for next generation sequencing using a multi-gene colorectal cancer panel.

Variable	Frequency (N = 16), N
**Age**	
< 40	7
41–50	3
>50	6
**Female**	8
**Location**	
Proximal	6
Distal	2
Rectal	7
Synchronous (hepatic and rectal)	1
**Personal history of cancer**	
Colorectal cancer	0
Breast cancer	1
Other cancers	0
**Family history**	
Any Lynch cancer	3
Colorectal cancer	2
Breast and duodenal cancers	1
Unspecified bowel cancer	1
**Histology**	
Adenocarcinoma	11
Mucinous adenocarcinoma	2
Signet ring cell carcinoma	3
**Other pathological characteristics**	
Synchronous tumours	1
Multiple colonic polyps	1
**MSI status**	
MSS	5
MSI-H	6
MSI-L	0
Unknown	5
**MMR deficiency**	
*MLH1* and *PMS2*	4
*MSH2* and *MSH6*	1
*MSH2* alone	1

**Table 4:** Confirmed and probable cases of inherited colorectal cancer syndromes in the cohort.

Age (Sex)	Cancer site	Family history	MMR Status	Affected gene	Nucleotide change	Protein change
*Lynch Syndrome*						
34(F)	Transverse colon	Father, suspected CRC, 40s. Sister colon, 42. Two aunts, colon, 30s & 72. Two cousins, unknown cancers.	MSI-H *MLH1* and *PMS2* deficient	Likely *MLH1*	unidentified	?
41(M)	Rectum	Brother and maternal aunt: unclear intestinal tumours	MMR proficient	*MLH1*	*c*.(1896+1_1897–1)_(*193_?*)del *	?
48(F)	Rectum	Unknown	*MSH2* deficient	*MSH2*	*c*.*2634G>A *	*p*.*Glu878 = *
***Other***						
54(F)	Rectum	Proband previous breast cancer, Sister–breast, 44 years. Father- duodenal. Mother- lung adenocarcinoma, non-smoker	Proficient	?	Unidentified	?
35(F)	Rectum	Mother–CRC 50 years	Proficient	?	Unidentified	?

### The frequency and patterns of *MLH1*, *MSH2* and *EPCAM* deletions

MLPA for *MLH1*, *MSH2* and *EPCAM* germline deletions was successful in 66 cases. One likely pathogenic deletion was identified, an *MLH1* Ex 17–19 deletion, i.e., NM-000249.3, *MLH1 c*. (1896+1_1897–1)_(*193_?*)del*, in a 41-year-old man with a rectal adenocarcinoma. The tumour was mismatch repair protein proficient and there was a history of unspecified intestinal cancer in his brother, and maternal aunt (Table 4). Deletions were also identified in two other cases, an *MSH2* ex 11 deletion, and an *MSH2* ex 6 deletion. These were small and considered likely false positive. This assertion was supported by the unremarkable phenotype in both cases.

### Summary of inherited colorectal cancer syndromes in the cohort

**Fig 2:**
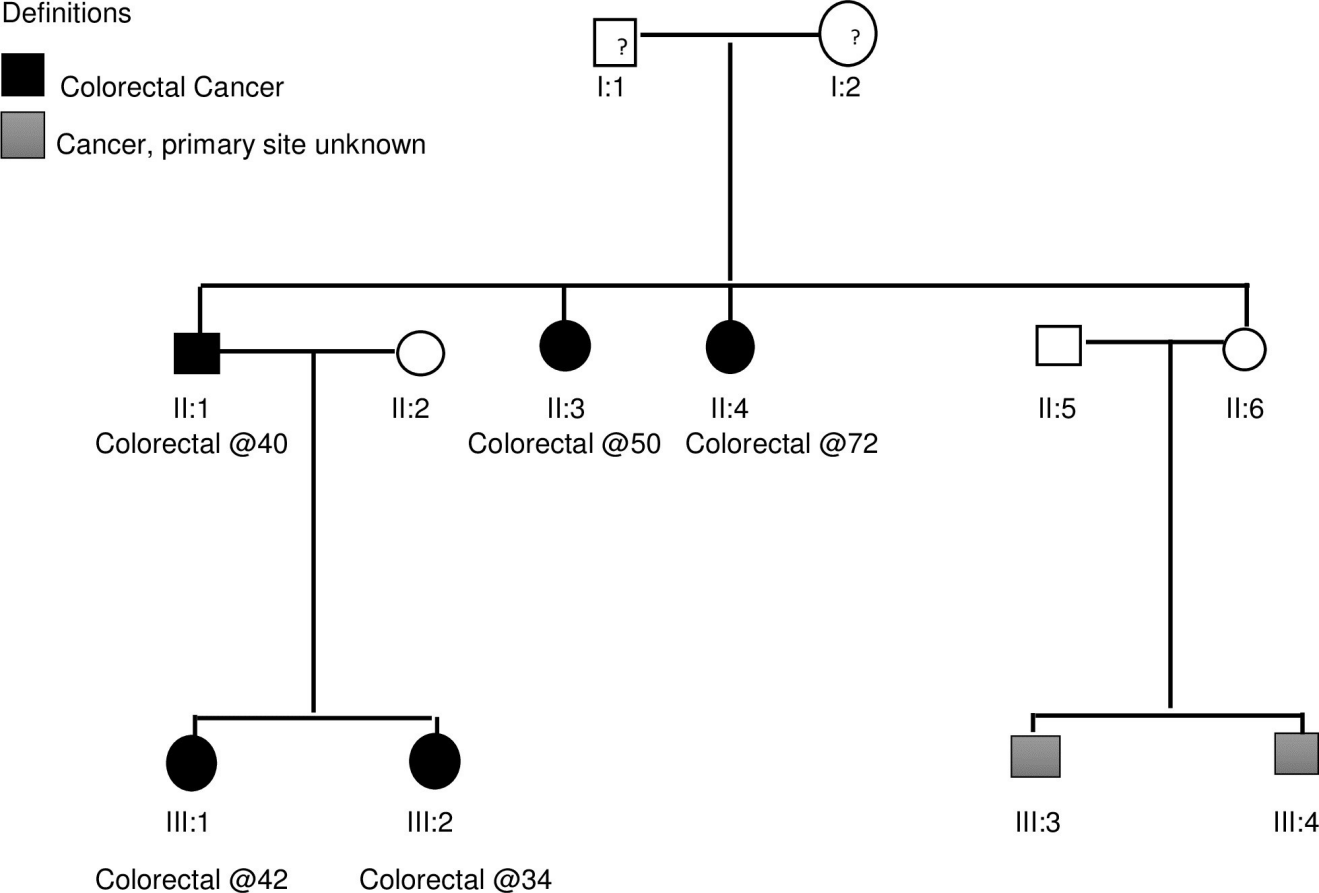
Pedigree analysis in a Lynch syndrome case meeting the Amsterdam II criteria, but with an unidentified *MLH1* or *PMS2* mutation.

There were 3 Lynch syndrome cases (Table 4) giving a prevalence of 3·3% (95% CI, 0·7–9·4). These were as follows: *MSH2*, *c*.*2634G>A* mutation, *MLH1 c*.(1896+1_1897–1)_(*193_?*)del *, and the case fulfilling the Amsterdam criteria. The individual fulfilling the Amsterdam criteria was a 34-year-old woman with a well-differentiated adenocarcinoma in the transverse colon, and a history of colorectal cancers in both 1^st^ and 2^nd^ degree relatives (Table 4 and Fig 2). The tumour was MSI-H, *MLH1* and *PMS2* deficient, and negative for *BRAF V600E*, but no pathogenic mutation was identified on next generation sequencing. The case fulfilled the Amsterdam II criteria, making a double somatic mutation as an explanation for the mismatch repair deficiency unlikely. There were two other cases with a family history highly suggestive of inherited colorectal cancer syndromes, but no mutations were identified (Table 4). They were both mismatch repair protein proficient, suggesting syndromes other than Lynch. A variant of unknown significance was identified in one of these individuals, a 54-year-old woman with a previous history of breast cancer. This was a missense mutation in *MSH6* (NM_000179.2; *c*.*124C>T; p*.*Pro42Ser*). [15]

Therefore, it can be reasonably concluded that, the minimum prevalence of inherited colorectal cancer syndromes in our population ranges from 3·3% (95% CI 0·7–9·4) to 5·6% (95% CI, 1·8–12·5).

## Discussion

This study aimed to establish the role of the established hereditary syndromes in colorectal cancer in sub-Saharan Africa, where the frequency of early-onset disease is disproportionately high. We concluded that up to 1 in 18 patients with colorectal cancer have an underlying high penetrance genetic predisposition. However, we found no evidence to support the hypothesis that the high frequency of early onset colorectal cancer in our population is associated with frequent germline mutations in the established high penetrance genes, particularly Lynch syndrome. This correlates with the axiom that, while most hereditary colorectal cancers occur at an early age, most early onset colorectal cancers are not hereditary.[16]

In our study, 3·3–5·6% of patients had hereditary colorectal cancers, which is similar to estimates from the traditional high incidence countries.[17] The prevalence of Lynch syndrome was also within the global range of 2–4%.[18] There were no germline pathogenic variants in the other known colorectal cancer susceptibility genes sequenced using the multi-gene panel. Thus, although there is a high rate of early onset colorectal cancer in our population, we found no convincing evidence of a correspondingly high frequency of familial cases. In contrast, recent evidence suggests that the prevalence of germline pathogenic variants in colorectal cancer susceptibility genes among young people is underestimated.[5, 19] Despite this, we are reasonably certain that the majority of the early onset colorectal cancers in our study were sporadic. It is possible that there are contributory environmental factors unique to this patient population; we have previously demonstrated a relationship between colorectal cancer and prior schistosomiasis.[3]

Our findings have implications for screening for Lynch syndrome in clinical practice in low resource settings. International guidelines recommend universal screening for mismatch repair deficiency of all colorectal cancers occurring below the age of 70 years.[11, 20] Universal screening is particularly suited to sub-Saharan Africa, where implementing phenotypic approach has unique challenges. The family history of cancers is masked by the high burden of infectious diseases, low life expectancy, low diagnostic rates and lack of population level knowledge of familial colorectal cancer.[21, 22] However, universal screening is impractical in sub-Saharan Africa, given the competing priorities for resources. While tools such as the Bethesda guidelines may be utilised, they are likely to have a low positive predictive value as a high proportion of colorectal cancer patients meet the criteria. A viable option for sub-Saharan Africa would be to lower the cut-off age for universal screening to 50 years–the Lynch syndrome cases identified in our study were all younger than 50 years. Our study also reaffirms the role of immunohistochemistry in screening for Lynch syndrome in clinical practice, and this is a low-cost option for low and medium income countries.[23]

In contrast, our findings suggest that *BRAF V600E* screening may not be useful in sub-Saharan Africa. The 3 cases that were MSI-H, *MLH1* deficient and *BRAF V600E* negative were all found to be sporadic after sequencing. The 4^th^ case had phenotypic features of Lynch syndrome, and the BRAF *V600E* result would have been inconsequential in clinical practice. A retrospective study in Ghana found no *BRAF* mutations in 88 unselected cases, despite a high proportion of MSI-H tumours.[9] Taken together, this suggests that BRAF mutations are uncommon in sporadic colorectal cancer in sub-Saharan Africa. The role of BRAF mutation, or *MLH1* hypermethylation promoter testing in African populations require further confirmation in a hypothesis driven study. An analogy can be made drawn with the Chinese population, where a low frequency of BRAF mutations was noted, and analysis for MLH1 hypermethylation was shown to be more efficient for triaging cases for further genetic analysis.[24] We did not evaluate specifically for *MLH1* hypermethylation in our study because the number of cases was too small to justify the added cost. Moreover, next generation sequencing was performed in all the cases that may have required *MLH1* hypermethylation analysis.

Our study had limitations which must be taken into account when interpreting the findings. First, we used a phenotype guided strategy to select cases for sequencing to reduce costs. Consequently, we may have missed some inherited colorectal cancer syndromes, and our findings must be regarded as the minimum possible prevalence. However, it is unlikely that we underestimated the prevalence of Lynch syndrome, which we screened for comprehensively using a combination of immunohistochemistry, MSI testing, MLPA, and next generation sequencing. Second, it is possible that we missed pathogenic germline variants in moderate penetrance colorectal cancer genes such as *ATM*, *SMAD3*, or high penetrance syndromes primarily associated with other cancers such as *BRCA1* and *BRCA2*, which were not part of our panel. For example, one of our participants, who had a prior history of breast cancer, and a family history of breast and gastrointestinal cancers could have a *BRCA 1* or *2* mutation. It is increasingly recognised that *BRCA 1* or *2* mutations are associated with colorectal cancer, although this may not be causal.[25] Third, not all early-onset cases were sequenced in our study, thus the findings in this sub-group must be considered exploratory. Fourth, case series of this nature may be subject to referral bias, particularly of young individuals and those with a positive family history of colorectal cancer. Our recruitment strategy minimised this by targeting all colorectal cancer patients seen at different levels of care within the catchment area. The similarity in the age distribution of the participants in our study, and the pattern described previously in sub-Saharan Africa is reassuring.[1] Finally, access to healthcare in Zimbabwe is uneven, and often affected by cultural and economic factors, and a significant proportion of gastrointestinal cancers are probably not identified. Thus, it is likely that our study missed some colorectal cancer cases in the study population.

However, this is the first prospective study to comprehensively evaluate the contribution, and patterns of inherited colorectal cancer syndromes in the poorly researched populations of sub-Saharan Africa. This study had comprehensive phenotypic data, and was based on an unselected population from both rural and urban areas. Thus, it can be considered representative of patients with colorectal cancer in sub-Saharan Africa. It forms a solid basis for larger studies, covering an even wider array of colorectal cancer susceptibility genes, and for context-specific clinical guideline development. As the immediate next step, it would be reasonable to consider performing whole exome sequencing for both tumour and germline tissue in individuals under the age of 50 years to more fully explore the involvement of other genes in early onset of colorectal cancers in our population.

To conclude, at least 1 in 30 patients with colorectal cancer in our population have Lynch syndrome. Identifying cases of inherited colorectal cancer and instituting screening among family members can have a quantifiable impact on the incidence in low prevalence countries. The use of a detailed family history, complemented by screening for Lynch syndrome using immunohistochemistry should be encouraged in these countries, where genomic technologies are unavailable in clinical practice. Ultimately, the goal should be to screen all young patients for Lynch syndrome in these countries using immunohistochemistry, and to intensify research into the causes of colorectal cancer in this demographic group. Our study demonstrates the feasibility of next generation sequencing in low- and medium-income countries, and hopefully these will eventually cascade into clinical practice as the costs continue to fall. Lastly, the majority of colorectal cancers in young people in Zimbabwe, and probably sub-Saharan Africa are not due to Lynch syndrome. This provides a unique opportunity for collaborative research into early onset colorectal cancer, which is an emerging global challenge.

## Supporting information

S1 File Dataset for the study on the hereditary basis of colorectal cancers in Zimbabwe. (XLSX)
